# Supplementary figures and images for: A Modified Actin (Gly65Val Substitution) Expressed in Cotton Disrupts Polymerization of Actin Filaments Leading to the Phenotype of Ligon Lintless-1 (Li1) Mutant
Source: Int J Mol Sci. 2021 Mar 16;22(6):3000. doi: 10.3390/ijms22063000 (PMC7998759; doi:10.3390/ijms22063000)

S fig1

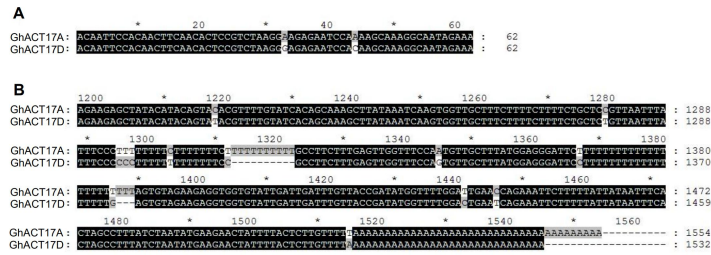

S fig2

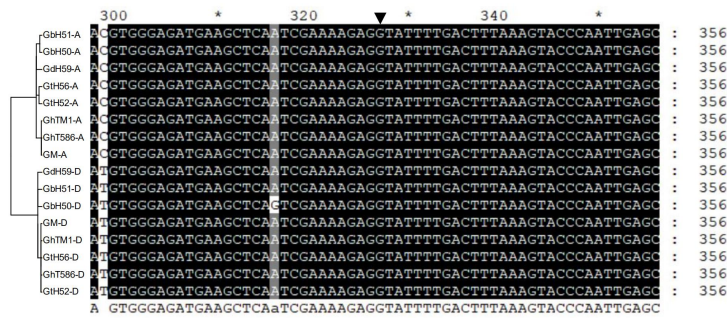

S fig3

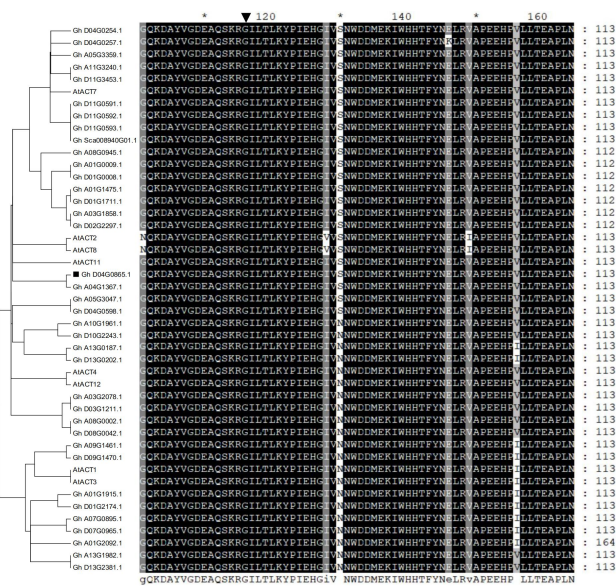

S fig4

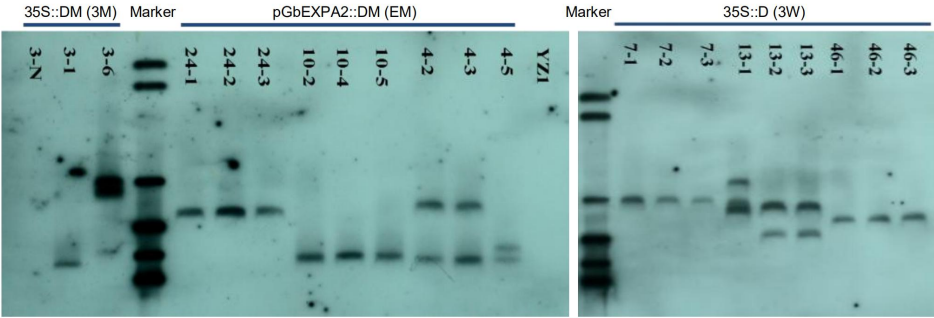

S fig5

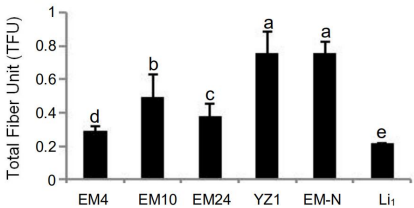

S fig6

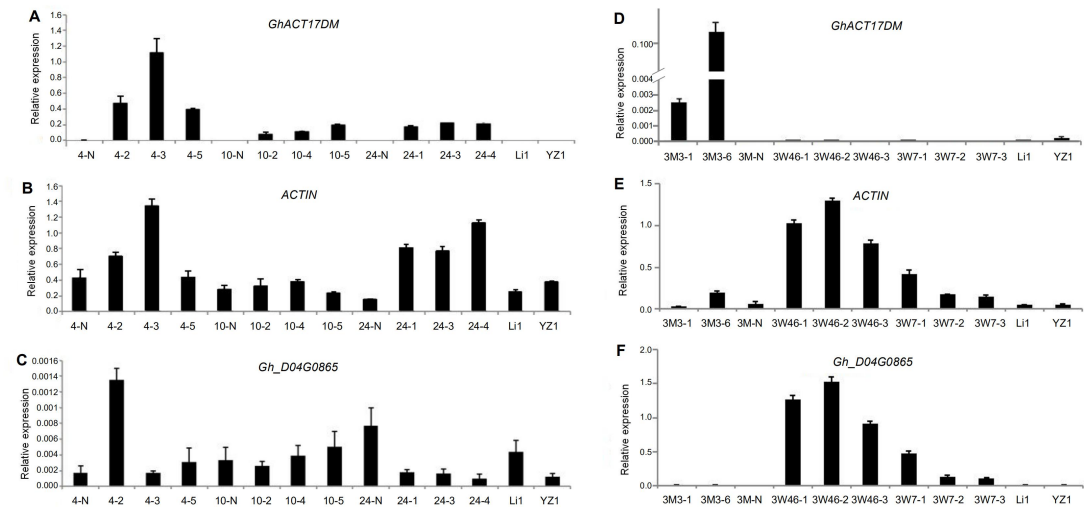

Supplement: Supplementary file 1 [file ijms-22-03000-s001.zip › Supp data/Supp Figs.pdf]
